# Treatment with specific soluble factors promotes the functional maturation of transcription factor-mediated, pancreatic transdifferentiated cells

**DOI:** 10.1371/journal.pone.0197175

**Published:** 2018-05-16

**Authors:** Hiroaki Motoyama, Akira Kobayashi, Takahide Yokoyama, Akira Shimizu, Hiroshi Sakai, Tsuyoshi Notake, Kentaro Fukushima, Shin-ichi Miyagawa

**Affiliations:** First Department of Surgery, Shinshu University School of Medicine, Matsumoto, Nagano, Japan; International University of Health and Welfare School of Medicine, JAPAN

## Abstract

Pancreatic lineage-specific transcription factors (TFs) display instructive roles in converting adult cells to endocrine pancreatic cells through a process known as transdifferentiation. However, little is known about potential factors capable of accelerating transdifferentiation following transduction to achieve the functional maturation of transdifferentiated cells. In this study, we demonstrated, using adult liver-derived progenitor cells, that soluble factors utilized in pancreatic differentiation protocols of pluripotent stem cells promote functional maturation of TFs-mediated transdifferentiated cells. Treatment with an N2 supplement in combination with three soluble factors (glucagon-like peptide-1 [GLP-1] receptor agonist, notch inhibitor, and transforming growth factor-β [TGF-β] inhibitor) enhanced liver-to-pancreas transdifferentiation based on the following findings: i) the incidence of c-peptide-positive cells increased by approximately 1.2-fold after the aforementioned treatment; ii) the c-peptide expression level in the treated cells increased by approximately 12-fold as compared with the level in the untreated cells; iii) the treated cells secreted insulin in a glucose-dependent manner, whereas the untreated cells did not; and iv) transplantation of treated-transdifferentiated cells into streptozotocin-induced immunodeficient diabetic mice led to the amelioration of hyperglycemia. These results suggest that treatment with specific soluble factors promotes the functional maturation of transdifferentiated cells. Our findings could facilitate the development of new modalities for cell-replacement therapy for patients with diabetes.

## Introduction

Allogeneic islet transplantation offers a minimally invasive option for β-cell replacement in patients with type 1 diabetes (T1D). However, the widespread application of this treatment is limited because of the scarcity of donor tissue and health concerns associated with the chronic use of immunosuppressive drugs in the recipient. To overcome these limitations, efforts have been focused on insulin-producing cells derived *in vitro* from human pluripotent stem cells [1, 2]. In particular, recent advances in the use of human induced pluripotent stem cells (hiPSCs) have enabled the production of functional insulin-producing cells with many characteristics that closely resemble those of bona fide β cells *in vitro* [2]. This success marks the beginning of a novel transplantation treatment for diabetes using patient-derived hiPSCs that could eliminate the need for immunosuppression. However, since the engraftment of transplanted islets has never been satisfactory in T1D patients (with an insulin independence rate of less than 50% at 3 years after transplantation [3]), the engraftment potential might be a rate-limiting step in hiPSCs-based cell therapy, despite the fact that cell transplantation is indispensable for hiPSCs-based cell therapy. Furthermore, another concern is that patients with T1D may not benefit from personalized hiPSCs-derived β-cells because of autoimmune rejection of the reconstituted β-cells. Hence, for clinical application of hiPSC-based cell therapy, development of an immunoprotective method (such as macro- or micro-encapsulation [4]) is required, in parallel with further improvements in pancreatic induction protocols.

Alternatively, transdifferentiation is a process in which one adult cell type is directly converted into an alternate cell type with a different function [5]. The ectopic expressions of lineage-specific transcription factors (TFs) have been suggested to display instructive roles in these processes [6, 7]. Unlike hiPSCs, although the inducibility of TFs-mediated transdifferentiation varies according to the TFs that are being transduced, transdifferentiation occurs within a few days once the appropriate TFs have been administered [8, 9]. Furthermore, the potential advantage of TFs-mediated transdifferentiation is that functional transdifferentiated cells can be induced *in situ* in targeted tissues. Thus, TFs-mediated transdifferentiation could enable the establishment of a regenerative therapeutic modality without the need for cellular transplantation.

Recently, Baeyens et al. showed that it is possible to induce pancreatic exocrine-to-endocrine transdifferentiation artificially using the transient administration of epidermal growth factor and ciliary neurotrophic factor [10]. Although this approach is expected to establish a transgene-free cellular transdifferentiation strategy, it simultaneously raises the possibility that there are specific soluble factors that act synergistically with transduced TFs to promote cellular transdifferentiation. However, little attention has been paid to exploring factors that are suitable for TFs-mediated transdifferentiation.

In the current study, we analyzed the inducibility of TFs-mediated cellular transdifferentiation using portal branch ligation-stimulated hepatic cells (PBLHCs), which were bipotent hepatic progenitor cell clones that were isolated from the portal branch-ligated liver lobes of adult mice at our institution [11], by culturing the cells in a serum-free medium containing specific soluble factors using a three-dimensional (3D) cellular aggregation technique. We found that treatment with an N2 supplement in combination with three soluble factors (glucagon-like peptide-1 [GLP-1] receptor agonist, notch inhibitor, and transforming growth factor-β [TGF-β] inhibitor) promote functional maturation of TFs-mediated transdifferentiated cells. This result could lead to a novel therapy modality for diabetes.

## Materials and methods

### Portal branch ligation-stimulated hepatic cells

The portal branch ligation-stimulated hepatic cells (PBLHCs) were bipotent hepatic progenitor cell clones that were isolated from the portal branch-ligated liver lobes of adult mice at our institution. The isolation of PBLHCs was performed as described previously [11]. Briefly, 8- to 12-week-old C57Bl/6 J mice (CLEA Japan, Tokyo, Japan) were used for the portal vein ligation experiments. The mice were sacrificed on day 7 after portal vein ligation. The animal protocol was approved by the Animal Experimentation Committees of Shinshu University. Isolated liver cells were prepared using a 2-step collagenase perfusion technique, as described previously [12]. The non-parenchymal cell fraction was plated in a laminin-coated plate (BD Biosciences, Franklin Lakes, NJ) and cultured in William’s E medium supplemented with 5% fetal bovine serum (Sigma-Aldrich, St. Louis, MO), 1× penicillin/streptomycin/glutamine (Thermo Fisher Scientific, Waltham, MA), recombinant mouse epidermal growth factor (20 ng/mL; R&D Systems, Minneapolis, MN), nicotinamide (10 mmol/L; Sigma-Aldrich), 1× Insulin–Transferrin–Sodium Selenite Supplement (Roche Diagnostics, Mannheim, Germany), and dexamethasone (0.1 uM; Sigma-Aldrich) with a humidified 5% CO_2_/95% air atmosphere at 37°C. Aforementioned culture medium was denoted as serum-containing medium (SCM) in this context. After 2–4 months, the proliferation of epithelial-like colony-forming subpopulations was observed, and the cells were cloned using the limiting dilution method, according to the procedure described by Park et al. [13]. We named these colony-forming cells as PBLHCs, and the cells were used in subsequent experiments as a possible source of TFs-mediated cellular transdifferentiation.

### βTC6 cells

Mouse βTC6 cells[14], purchased from ATCC, were maintained in Dulbecco’s Modified Eagle’s medium (Thermo Fisher Scientific) supplemented with 15% fetal bovine serum (Sigma-Aldrich), 1× penicillin/streptomycin/glutamine (Thermo Fisher Scientific), and 1× MEM Non-Essential Amino Acids Solution (Thermo Fisher Scientific). All cells were grown at 37°C in a humidified air incubator in the presence of 5% CO_2_.

### Adenovirus vectors

Recombinant adenoviruses expressing pancreatic TFs of mouse together with nGFP were kindly provided by Douglas Melton. The details of the plasmids were as follows: pAd Pdx1-I-nGFP (Addgene plasmid # 19411), pAd 2B Ngn3-I-nGFP (# 19410), pAd NeuroD-I-nGFP (# 19414), and pAd MafA-I-nGFP (# 19412) [7]. Adenovirus constructed for the 2A peptide-mediated polycistronic expression of four genes (Pdx1, Ngn3, MafA, and monomeric cherry fluorescent protein [mCherry]) was also provided by Addgene as a generous gift from Qiao Zhou (pAd-M3cherry, # 61041) [15]. pAd-M3cherry was denoted as pAd-PNMcherry (PNMc) in this context to clarify the difference in transduced TFs. The viral titers were estimated using the Adeno-X qPCR Titration Kit (Clontech) according to the manufacturer’s protocol. The optimal MOI was determined as a titer achieving an infection rate of above 80% in cultured PBLHCs at 24 hours after infection with the manual counting of nGFP-positive cells. Since mCherry fluorescence was hardly visible at 24 hours after infection, the experiments were conducted using a viral titer similar to that of the nGFP expression vector. The MOIs of the viruses used in the experiments were as follows: pAd Pdx1-I-nGFP, 5 MOI; pAd 2B Ngn3-I-nGFP, 5 MOI; pAd NeuroD-I-nGFP, 5 MOI; pAd MafA-I-nGFP, 50 MOI; and pAd-M3cherry, 5 MOI.

### Serum-free differentiation medium containing specific compounds (SFDM) for *in vitro* transdifferentiation

To analyze whether liver-to-pancreas direct transdifferentiation is facilitated by specific compounds, we prepared a serum-free differentiation medium containing specific soluble factors (SFDM). SFDM was prepared by referring to the protocols for generating pancreatic endocrine cells from hiPSCs/human embryonic stem cells (hESCs), especially the molecules used in the process of endocrine lineage commitment [1, 16, 17]. In brief, SFDM was a medium supplemented with below-mentioned molecules and supplements for basal medium mainly composed of IMDM and Hamm’s F12 (IH medium). The selected molecules and supplements were as follows: epidermal growth factor (EGF), exendin-4 (GLP-1 receptor agonist), forskolin (adenylate cyclase activator), L685,458 (notch inhibitor), nicotinamide, noggin (inhibitor of bone morphogenetic protein [BMP] pathway), SB431542 (inhibitor of TGF-β type 1 receptor), N2, and B27 supplement. The detailed formulations of SFDM are described in the Table 1.

**Table 1:** Formulation of serum free differentiation medium containing specific compounds (SFDM).

Reagent	Company	Working concentration
Basal medium (IH medium)		
IMDM	Thermo Fisher Scientific	75%
Ham's F12	Thermo Fisher Scientific	25%
Penicillin/Streptomycin/Glutamine	Thermo Fisher Scientific	1%
Ascorbic acid	Sigma-Aldrich	50 μg/mL
BSA	Sigma-Aldrich	0.05%
Glucose	Wako	40 mmol/L
Molecules (mixture of 7 molecules, M7)		
Epidermal growth factor	R&D Systems	50 ng/mL
Exendin-4	Sigma-Aldrich	5 nmol/L
Forskolin	Wako	10 μmol/L
Nicotinamide	Sigma-Aldrich	10 mmol/L
Noggin	TONBO Bioscience	50 ng/mL
L685,458	Peptide Institute	1 μmol/L
SB431542	Cayman Chemical	6 μmol/L
Supplements		
B27 supplement	Thermo Fisher Scientific	1%
N2 supplement	Thermo Fisher Scientific	1%

EGF, epidermal growth factor; BSA, bovine serum albumin.

### Culture formats for *in vitro* transdifferentiation

PBLHCs obtained 1 day after the transduction of TFs with adenovirus vector were trypsinized and re-seeded either for adherent culture (AC) (2.5 × 10^4^ cells/cm^2^) or suspension culture (SC) (5.0 × 10^5^ cells/mL). To form cellular aggregates in SC, the detached cells were aliquoted into 15-mL conical tubes and preincubated statically in a humidified atmosphere of 5% CO_2_/95% air at 37°C for 2 hours. Then, the cells were re-seeded into 24-well ultra-low attachment culture dishes (Corning, Corning, NY) with appropriate mediums.

### Semi-quantitative RT-PCR and real-time quantitative RT-PCR (qRT-PCR)

Total RNA was isolated from at least triplicate samples in three independent experiments using an RNeasy plus mini kit (Qiagen, Venlo, Netherlands), followed by cDNA synthesis using standard protocols. Semi-quantitative RT-PCR was performed as described previously [9]. The primer sequences used for semi-quantitative RT-PCR are available upon request. qRT-PCR was performed in triplicate using Eppendorf Mastercycler ep Gradient S (Eppendorf, Hamburg, Germany) with the TaqMan Gene Expression Assay (Applied Biosystems, Waltham, MA). The cDNA samples subjected to qRT-PCR were synthesized from 500 ng of total RNA using PrimeScript RT-PCR Kit (TaKaRa Bio). The assay mixes that were analyzed were as follows: insulin I (Mm01259683_g1), insulin II (Mm00731595_gH), glucagon (Mm00801714_m1), somatostatin (Mm00436671_m1), pancreatic polypeptide (Mm01250509_g1), proprotein convertase subtilisin/kexin type 1 (Mm00479023_m1), proprotein convertase subtilisin/kexin type 2 (Mm00500981_m1), ATP-binding cassette, sub-family C (CFTR/MRP), member 8 (Mm00803450_m1), solute carrier family 2 (facilitated glucose transporter), member 2 (Mm00446229_m1), pancreatic and duodenal homeobox 1 (Mm00435565_m1), neurogenin 3 (Mm00437606_s1), neurogenic differentiation 1 (Mm01946604_s1), v-maf musculoaponeurotic fibrosarcoma oncogene family, protein A (avian) (Mm00845206_s1), paired box 4 (Mm01159036_m1), paired box 6 (Mm00443081_m1), ISL1 transcription factor, LIM/homeodomain (Mm00517585_m1), aristaless related homeobox (Mm00545903_m1), NK6 homeobox 1 (Mm00454961_m1), albumin (Mm00802090_m1), cytochrome P450, family 1, subfamily a, polypeptide 2 (Mm00487224_m1), CCAAT/enhancer binding protein (C/EBP), beta (Mm00843434_s1), transferrin (Mm00446715_m1), keratin 19 (Mm00492980_m1), alpha fetoprotein (Mm00431715_m1), and glyceraldehyde-3-phosphate dehydrogenase (Gapdh; Mm99999915_g1). The expression levels of each gene were normalized to those of Gapdh in the same samples.

### Measurement of intracellular content of c-peptide

The samples of infected PBLHCs were washed four times with PBS and then treated with acid-ethanol (0.18 mol/L hydrochloric acid in 95% ethanol) at 4°C overnight. The clear supernatants were used to assay the intracellular insulin and c-peptide contents, and the values obtained were normalized relative to the total protein content. The c-peptide contents were measured using an enzyme-linked immunosorbent assay (Mouse C-peptide ELISA Kit, Shibayagi, Gunma, Japan). The total protein content was measured using Protein Assay (Bio-Rad, Hercules, CA).

### Glucose-stimulated insulin secretion assay

Transdifferentiated cellular aggregates (almost 200 aggregates/assay) treated with SCM or SFDM were sampled. The aggregates were washed with Krebs-Ringer bicarbonate HEPES buffer (KRBH: 116 mmol/L NaCl, 4.7 mmol/L KCl, 2.5 mmol/L CaCl_2_, 1.2 mmol/L KH_2_PO_4_, 1.2 mmol/L MgSO_4_, 24 mmol/L HEPES, 25 mmol/L NaHCO_3_, and 0.1% BSA) and were then preincubated in low (2.5 mmol/L) glucose KRBH for 2 hours to remove residual insulin. Aggregates were washed two times in KRBH, incubated in low-glucose KRBH for 60 min, and the supernatant was collected. Then, the aggregates were washed two times in KRBH, incubated in high glucose (22.5 mmol/L) KRBH for 60 min, and the supernatant was collected again. The supernatant samples containing secreted insulin were processed using the Mouse Insulin ELISA Kit (Shibayagi).

### Immunohistochemical analysis

The infected PBLHCs treated with AC or SC were prepared individually for immunohistochemical analysis as follows: samples treated with AC were fixed with 4% paraformaldehyde/PBS (PFA/PBS) for 20 min and then permeabilized with 0.1% Triton X/PBS for 10 min at room temperature. The cellular aggregates formed by SC were collected and embedded in iPGell (Genostaff, Tokyo, Japan) according to the manufacturer’s recommendations. The embedded samples were fixed with 4% PFA/PBS overnight at room temperature and sectioned for histological analysis. Immunohistochemical staining was performed as previously described [9, 11]; the antibodies are detailed in Supplemental Table 1. Representative images were taken using an BZ-8000 Fluorescence Microscope (Keyence, Osaka, Japan) or Olympus BX60 Fluorescence Microscope (Olympus, Tokyo, Japan).

### Measurement of c-peptide-positive cells

The cellular aggregates formed by SC were dispersed into a single-cell suspension by incubation in TrypLE Express (Thermo Fisher Scientific) at 37°C, then 3 μL of the cell suspension at a concentration of 2.5 × 10^7^ cells/mL were smeared using Smear Gell (Genostaff), according to the manufacturer’s protocol. Subsequently, c-peptide immunostaining was performed, and the rate of c-peptide-positive cells was determined by manually counting 50 random fields at 200× magnification in 5 independent samples.

### Streptozotocin (STZ)-induced diabetic mouse model and transplantation analysis

Male 9- to 12-week-old BALB/cAJcl-nu/nu mice (CLEA Japan) were maintained under specific pathogen-free conditions at 22 ± 2°C with 12 hours of artificial lighting from 9 AM and were fed chow and water ad libitum. Diabetes was induced via a single intraperitoneal injection of STZ (160 μg/g in 0.01 mol/L citrate buffer, pH4.6). Non-fasting blood glucose levels were measured using a Medisafe Mini Glucometer (Terumo, Tokyo, Japan). The following week, mice with non-fasting blood glucose levels of 300–600 mg/dL on 2 consecutive days were considered to have diabetes. For the transplantation analysis, the mice underwent left renal subcapsular transplantation with cellular aggregates collected from 12 wells in a 24-well plate under anesthesia using a mixture of medetomidine (0.3 mg/kg; Wako Chemicals, Osaka, Japan), midazolam (4 mg/kg; Wako Chemicals) and butorphanol tartrate (5 mg/kg; Wako Chemicals) administered by intraperitoneal injection according to the institutional recommendation to obtain the desired levels of sedation, analgesia and skeletal muscle relaxation. The mice were housed in a temperature- and humidity-controlled room and allowed access to a commercial diet and water *ad libitum*. A left nephrectomy was performed in all the transplanted mice at 28 days after the transplantation. Blood glucose levels were recorded until 35 days after transplantation, then the mice were sacrificed by cervical dislocation under sodium pentobarbital anesthesia (100 mg/kg; Dainabot, Osaka, Japan). To compare the functionality of the transdifferentiated cells with that of cells actually having insulin-secreting ability, we also created the βTC6-transplanted model by the aforementioned anesthetic and surgical procedure (1.0 × 10^4^ cells/body, n = 5). Transplanted mice were sacrificed humanely to alleviate suffering under the following conditions:

i) sustained decrease of the body weight to 80% or lower than that recorded before the induction of diabetes, indicative of malnutrition.
ii) reduction of the non-fasting blood glucose levels to 60 mg/dL or lower, indicative of severe hypoglycemia.

### Ethics statements

All experiments using animals were approved by the Shinshu University Animal Care Committee (Approval Number: 270022) and all the experiments using animal were performed according to the guideline presented by the committee.

### Statistics

The results were expressed as the mean ± standard deviation (S.D.). A statistical analysis of the data among the groups was performed using a *t*-test, Mann–Whitney *U* test, or a one-way analysis of variance (ANOVA) followed by Dunnett’s test for multiple comparisons, as appropriate. The groups used as a control for each of the multiple comparisons were denoted as such. All the analyses were performed using JMP software version 10.0 (SAS Institute, Cary, NC). Differences of *P* < 0.05 were considered statistically significant.

## Results

### A triple combination of TFs efficiently induces pancreatic transdifferentiation *in vitro*

**Fig 1:**
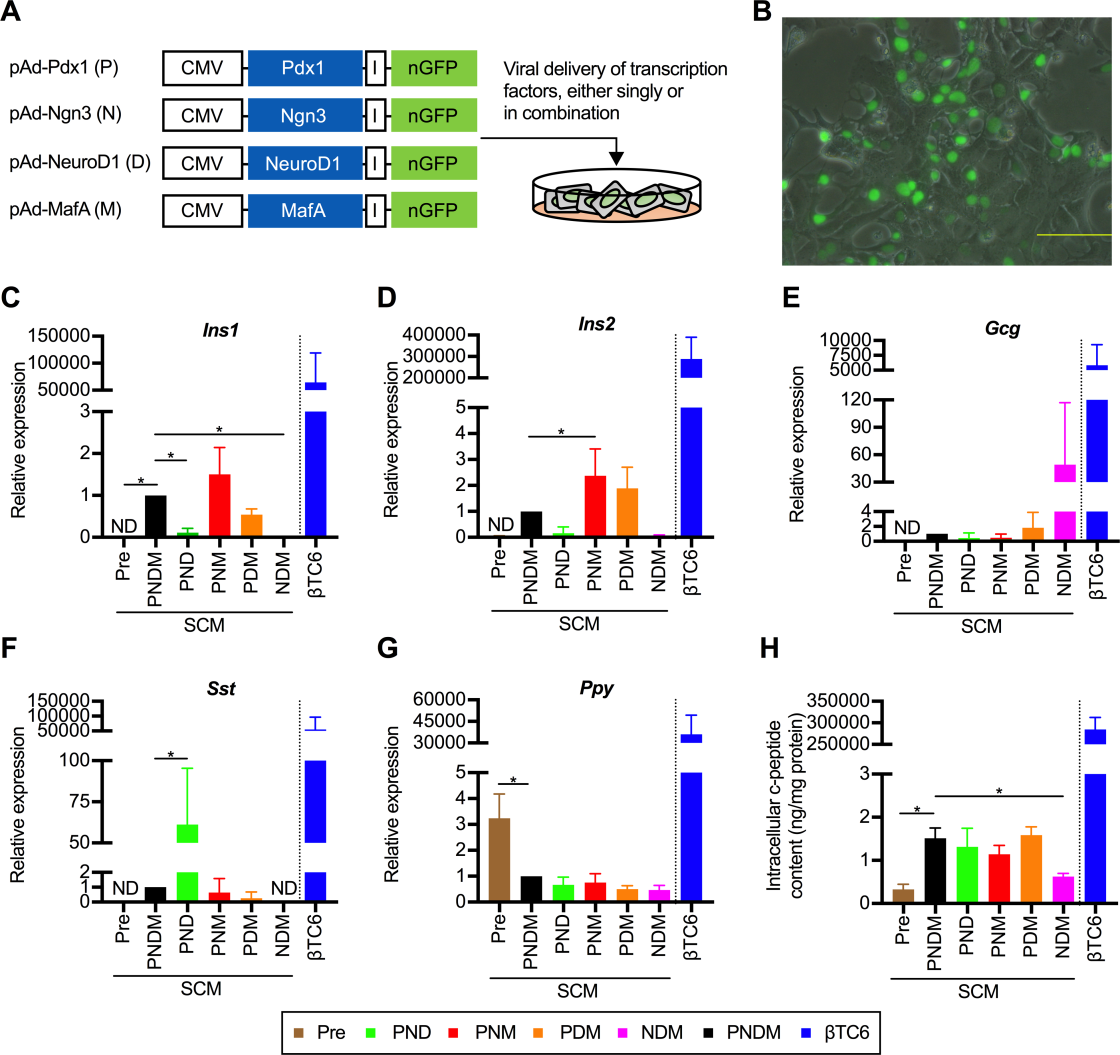
A triple combination of transcription factors (TFs) induces transdifferentiation into insulin-producing cells *in vitro*. (A) Schematic diagram of the experimental strategy used to optimize the transgene. Adenoviruses encoding bicistronic TFs and nuclear localized GFP (nGFP) linked by an IRES element (I) were used. Each vector containing genes encoding Pdx1, Ngn3, NeuroD1, and MafA is abbreviated as P, N, D, and M, respectively. The combination of P, N, D, and M means the co-infection of each vector. (B) Appearance of nGFP-positive cells on the day after transfection. Scale bar, 200 μm. (C, D, E, F and G) Quantitative real-time polymerase chain reaction (qRT-PCR) analyses stratified according to the combination of TFs that were used were performed using primer sets for *Ins1* (C), *Ins2* (D), *Gcg* (E), *Sst* (F) and *Ppy* (G). “Pre” represents PBLHCs without transfection. The data are presented as fold-changes in the gene expressions relative to the values in the cells transfected with PNDM (n = 4). *P < 0.05 (one-way ANOVA followed by Dunnett’s test. The values in the cells transfected with PNDM were used as the control.); error bars, S.D. (H) Intracellular c-peptide contents in the transfected cells (n = 4). *P < 0.05 (one-way ANOVA followed by Dunnett’s test. The values in the cells transfected with PNDM were used as control.); error bars, S.D. The results obtained from βTC6, a mouse insulinoma cell line, were excluded from all the statistical analyses, because of the marked deviations from other values. CMV, cytomegaloviral promoter; *Ins1*, insulin I; *Ins2*; insulin II; *Gcg*, glucagon; *Sst*, somatostatin; *Ppy*, pancreatic polypeptide.

Four adenovirus vectors co-expressing one of each of the examined TFs together with nuclear-localized GFP (nGFP), namely pAd-Pdx1, pAd-Ngn3, pAd-NeuroD1, and pAd-MafA, were prepared. The ratios of nGFP-positive cells at 24 hours after infection with the indicated vector at an optimal multiplicity of infection (MOI) were as follows: 94.1% ± 4.5% for pAd-Pdx1; 88.8% ± 7.6% for pAd-Ngn3; 88.6% ± 3.1% for pAd-NeuroD1; and 87.7% ± 4.2% for pAd-MafA. We defined a total of 15 patterns of gene transfer using the 4 aforementioned expression vectors as follows: single factor (4 patterns), double combination (6 patterns), triple combination (4 patterns), and quadruple combination (1 pattern) (Fig 1A). On the day after viral infection, numerous nGFP-positive cells appeared (Fig 1B). Immunostaining of the co-infected PBLHCs revealed the nuclear localization of each ectopically expressed TF and nGFP (S1A and S1B Fig). At 3days after infection, selective increases in the mRNA levels of insulin I (*Ins1*) and insulin II (*Ins2*) were observed in the cells that had been infected with a triple combination of adenovirus (Pdx1 and MafA, with either Ngn3 or NeuroD1) in a semiquantitative RT-PCR analysis (S1C and S1D Fig). In the qRT-PCR analyses, the insulin gene expression levels (*Ins1* and *Ins2*) were the most enhanced in the cells expressing the triple combination of Pdx1, Ngn3 and MafA (PNM) (Fig 1C and 1D). In particular, the expression of *Ins2* was significantly enhanced (Fig 1D) as compared to that in the cells expressing the quadruple combination of Pdx1, Ngn3, NeuroD1and MafA (PNDM). Although no significant intergroup differences in the expression of the glucagon gene (*Gcg*) were observed, *Gcg* expression was most enhanced in the cells expressing the triple combination of Ngn3, Neuro D1 and MafA (NDM) (Fig 1E). Expression of somatostatin (*Sst*) was the highest in the cells expressing the triple combination of Pdx1, Ngn3 and NeuroD1 (PND) (Fig 1F). Expression of pancreatic polypeptide (*Ppy*) was the highest in the non-transgenic control, and the expression levels were decreased in all the transgenic groups (Fig 1G). The intracellular c-peptide contents were similar among the cells transfected with triple combinations (PND, PNM and PDM [Pdx1, NeuroD1, and MafA]) as compared with those in the cells transfected with the quadruple combination (Fig 1H). Although the relative expression levels of *Ins1*, *Ins2*, and c-peptide protein in the PBLHCs expressing PNM were much lower as compared to those in the βTC6 cells (1/43000^th^, 1/120000^th^, and 1/250000^th^, respectively), the combination of Pdx1, Ngn3, and MafA was considered as the optimal for inducing selective transdifferentiation of the PBLHCs to insulin-producing cells. Therefore, we used this combination of TFs in subsequent analyses.

### Treatment with SFDM and 3D culture are necessary for efficient transdifferentiation

**Fig 2:**
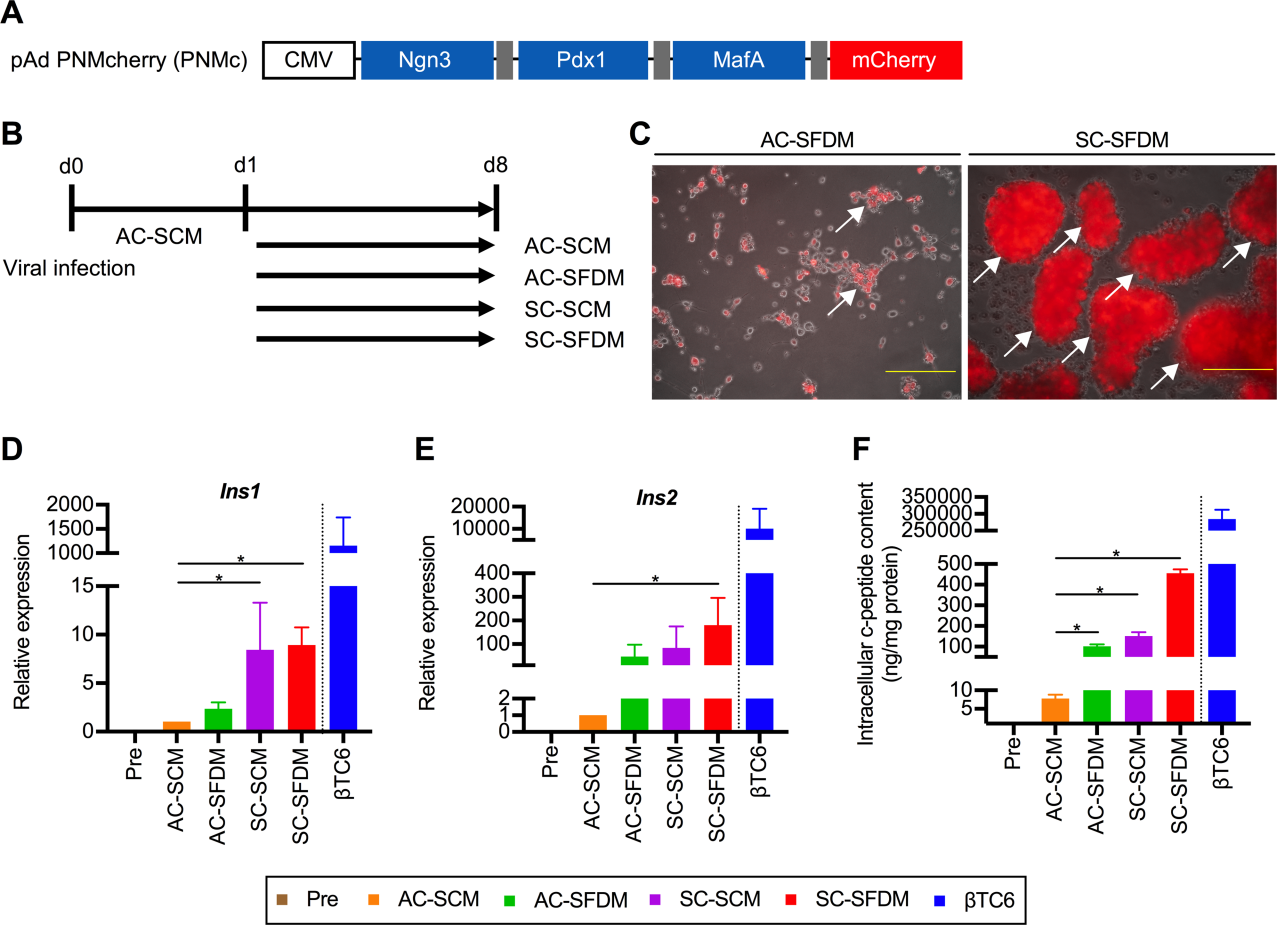
The assessment of the synergistic effects between a 3D culture system and serum-free differentiation medium for cellular transdifferentiation. (A) Adenoviral constructs for the polycistronic expression of three transcription factors (TFs). Dark gray bar, 2A peptide. (B) Schematic diagram of the procedures used to analyze the differences among culture systems for TFs-mediated transdifferentiation. (C) Representative microscopic images of infected cells treated with serum-free differentiation medium (SFDM) in two different culture formats. Arrow, cellular aggregate. Scale bars, 200 μm. (D and E) The analyses of insulin gene expressivity based on the difference in culture system. The expression of insulin I (*Ins1*: D) and insulin II (*Ins2*: E) was analyzed by quantitative real-time polymerase chain reaction (qRT-PCR) (n = 4). *P < 0.05 (one-way ANOVA followed by Dunnett’s test. The values in the cells treated with AC-SCM were used as control); error bars, S.D. (F) Intracellular c-peptide contents in infected cells (n = 4). *P < 0.05 (one-way ANOVA followed by Dunnett's test. The value of cells treated with AC-SCM was used as a control.); error bars, S.D. The results obtained from the βTC6 cells were excluded from all the statistical analyses, because of the marked deviations from other values. CMV, cytomegalovirus promoter; mCherry, monomeric cherry fluorescent protein; AC-SCM, adherent culture with serum-contained medium; AC-SFDM, adherent culture with serum-free differentiation medium; SC-SCM, suspension culture with serum-contained medium; SC-SFDM, suspension culture with serum-free differentiation medium.

Next, we analyzed whether cellular transdifferentiation is affected by the difference of culture medium. Simultaneously, we analyzed the differences in culture format, that is, the difference between an adherent culture (AC) and a suspension culture (SC). Since the expression levels of individual TFs may differ in cases with the co-infection of multiple vectors, we prepared another adenovirus vector to mediate the polycistronic expression of the 3 TFs and mCherry, namely PNMc (Fig 2A). We cultured the infected PBLHCs using 4 different culture systems: an AC with SCM (AC-SCM), an AC with SFDM (AC-SFDM), a SC with SCM (SC-SCM), and a SC with SFDM (SC-SFDM) (Fig 2B). Fig 2C shows representative microscopic images of the infected PBLHCs treated with SFDM in the two different culture formats. The SC cells had formed aggregates on the day after re-seeding, but no morphological changes were observed until day 8. In contrast, the AC cells first formed monolayers, and the cell number then gradually decreased. On day 8, although the AC cells had also formed aggregates, the aggregates were smaller than those of the SC cells. To examine the differences in inducibility among the 4 different culture systems, we first examined the expressions of insulin genes under the culture conditions (Fig 2D and 2E). The insulin gene expressions were markedly increased in the SC-SFDM-treated cells. In particular, the *Ins2* expression level in the SC-SFDM cells was increased by 180-fold, compared with that in the AC-SCM cells. Differences in the insulin-producing ability of the transfected cells depending on the culture systems were also analyzed based on quantitative evaluation of the c-peptide protein level. As a result, the intracellular contents of c-peptide in the SC-SFDM cells were found to be significantly elevated among all 4 culture systems. The amount of intracellular c-peptide in the SC-SFDM cells was prominent, with a 60-fold increase compared with the amount in AC-SCM cells (Fig 2F). The relative expression levels of *Ins1*, *Ins2*, and c-peptide protein in the PNM-transfected PBLHCs cultured in SC-SFDM were 1/129^th^, 1/56^th^, and 1/626^th^, respectively, as compared with those in the βTC6 cells. These results suggested that both the utilization of SFDM and a 3D culture system contributed to efficient transdifferentiation.

### Comprehensive analyzes about the effects of soluble factors on transdifferentiated cells

**Fig 3:**
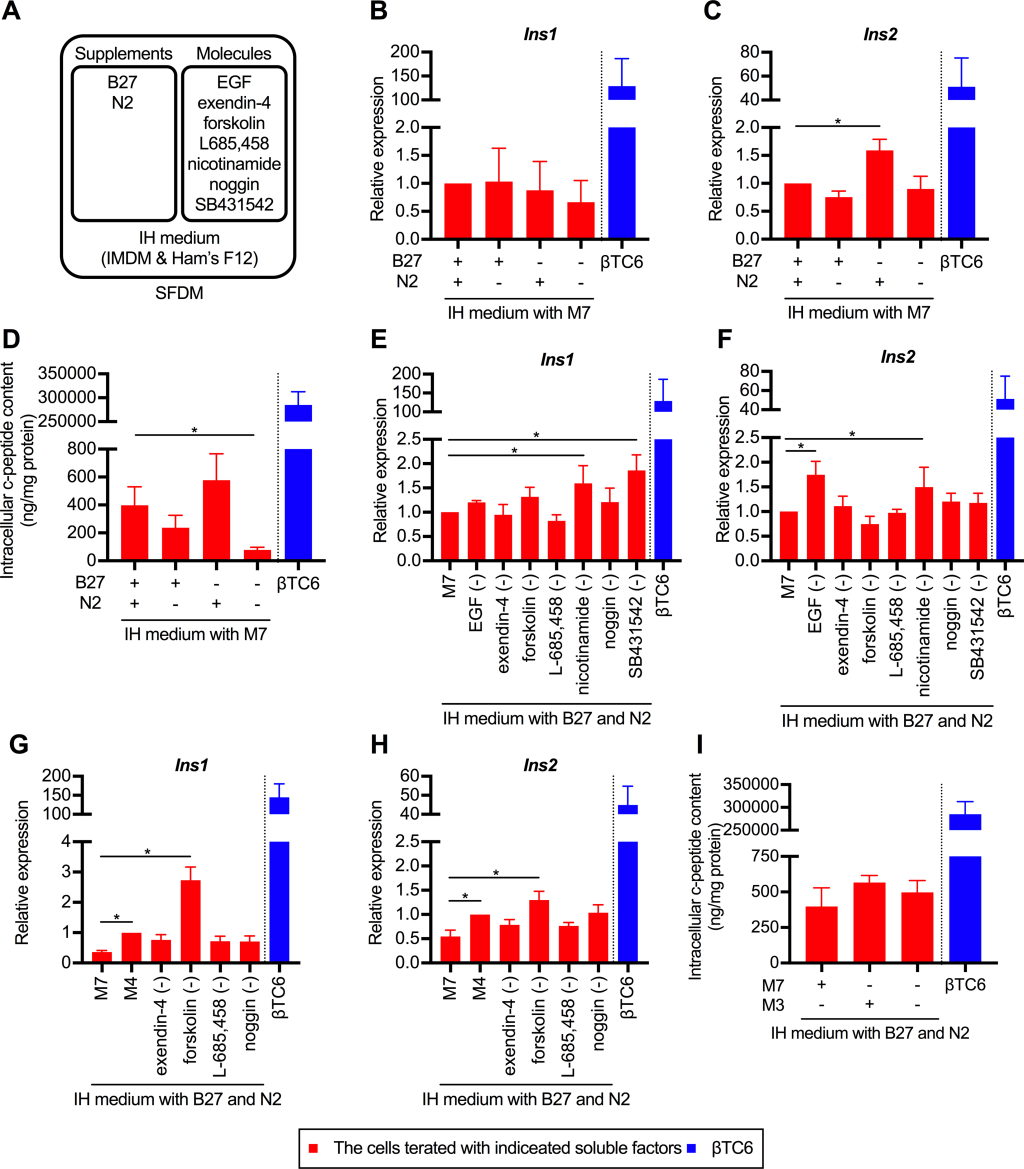
Comprehensive analyzes about extracellular environment of transdifferentiated cells. (A) Schematic diagram of the formulation of SFDM. (B and C) The expression of *Ins1* (B) and *Ins2* (C) of infected cells treated with indicated supplements. The data are presented as fold-changes in the gene expression levels relative to the values in the cells treated with N2 and B27 supplement (n = 4). IH medium indicates the basal medium composed of IMDM and Hamm’s F12. The precise composition is shown in Table 1. *P < 0.05 (one-way ANOVA followed by Dunnett's test. The value of cells treated with N2 and B27 supplements was used as a control.); error bars, S.D. (D) Differences in the intracellular c-peptide contents depending on the supplements added (n = 4). The values in the cells treated with N2 and B27 supplement were used as control. *P < 0.05 (one-way ANOVA followed by Dunnett’s test); error bars, S.D. (E, F, G and H) Influence on expression of insulin gene by individually removing seven molecules contained in SFDM. The *Ins1* (E and G) and *Ins2* (F and H) gene expressivity was analyzed when each factor was individually removed. M7 contained epidermal growth factor (EGF), exendin-4, forskolin, L685,458, nicotinamide, noggin, and SB431542. M4 contained exendin-4, forskolin, L685,458, and noggin. The data are presented as fold-changes in the gene expression levels relative to the values in the cells treated with M7 (E, F) or M4 (G, H) (n = 4). *P < 0.05 (one-way ANOVA followed by Dunnett's test. The value of cells treated with M7 [E, F] or M4 [G, H] was used as a control.); error bars, S.D. (I) Differences in the intracellular c-peptide contents depending on the supplements added (n = 4). The values in the cells treated with M7 were used as control. M3 contained exendin-4, L685,458 and noggin. *P < 0.05 (one-way ANOVA followed by Dunnett’s test. The values in the cells treated with M7 were used as control); error bars, S.D. The results obtained from the βTC6 cells were excluded from all the statistical analyses, because of the marked deviations from other values. B27, B27 supplement; N2, N2 supplement.

Based on the result that the induction of transdifferentiation is promoted by a serum-free 3D culture, we further investigated the effects of soluble factors on transdifferentiated cells. A unified 3D culture format was used. Since the factors added to SFDM were categorized as either a supplement composed of several factors (B27 and N2 supplement) or a single molecule (EGF, exendin-4, forskolin, L685,458, nicotinamide, noggin, and SB431542) (Table 1 and Fig 3A), we analyzed them individually. Firstly, we analyzed the necessity of B27 and N2 supplements. As shown in Fig 3B and 3C, *Ins2* gene expression was significantly increased in the cells treated without the B27 supplement. As for the intracellular c-peptide contents, the highest c-peptide level was observed in the cells treated with N2 supplement alone, although the differences were not statistically significant (Fig 3D). These result indicated that the addition of B27 supplement was not essential for the induction of cellular transdifferentiation. Next, we analyzed the necessity of molecules added to SFDM. We designated the mixture of the 7 aforementioned molecules as M7 (mixture of 7 molecules) and compared the expression levels of insulin genes when each molecule was individually removed. As a result, insulin gene expression was significantly increased, compared with the addition of M7, by the removal of EGF, nicotinamide, or SB431542 (Fig 3E and 3F). We further designated the mixture of exendin-4, forskolin, L685, 458, and noggin as M4 (mixture of 4 molecules) and conducted a similar analysis. As a result, the expression levels of both *Ins1* and *Ins2* were significantly increased by the removal of forskolin, whereas the addition of M7 significantly decreased the expression levels of both insulin genes, as compared to addition of M4 (Fig 3G and 3H). The intracellular c-peptide content was also higher in the cells treated with exendin-4, L-685,458 or noggin, than in the cells treated with M7. These findings indicated that among the seven molecules added to SFDM, exendin-4, L-685,458 and noggin (a mixture of 3 molecules, M3) were important for enhancing insulin gene expression.

### Simultaneous administration of N2 and M3 accelerated functional maturation of transdifferentiated cells

**Fig 4:**
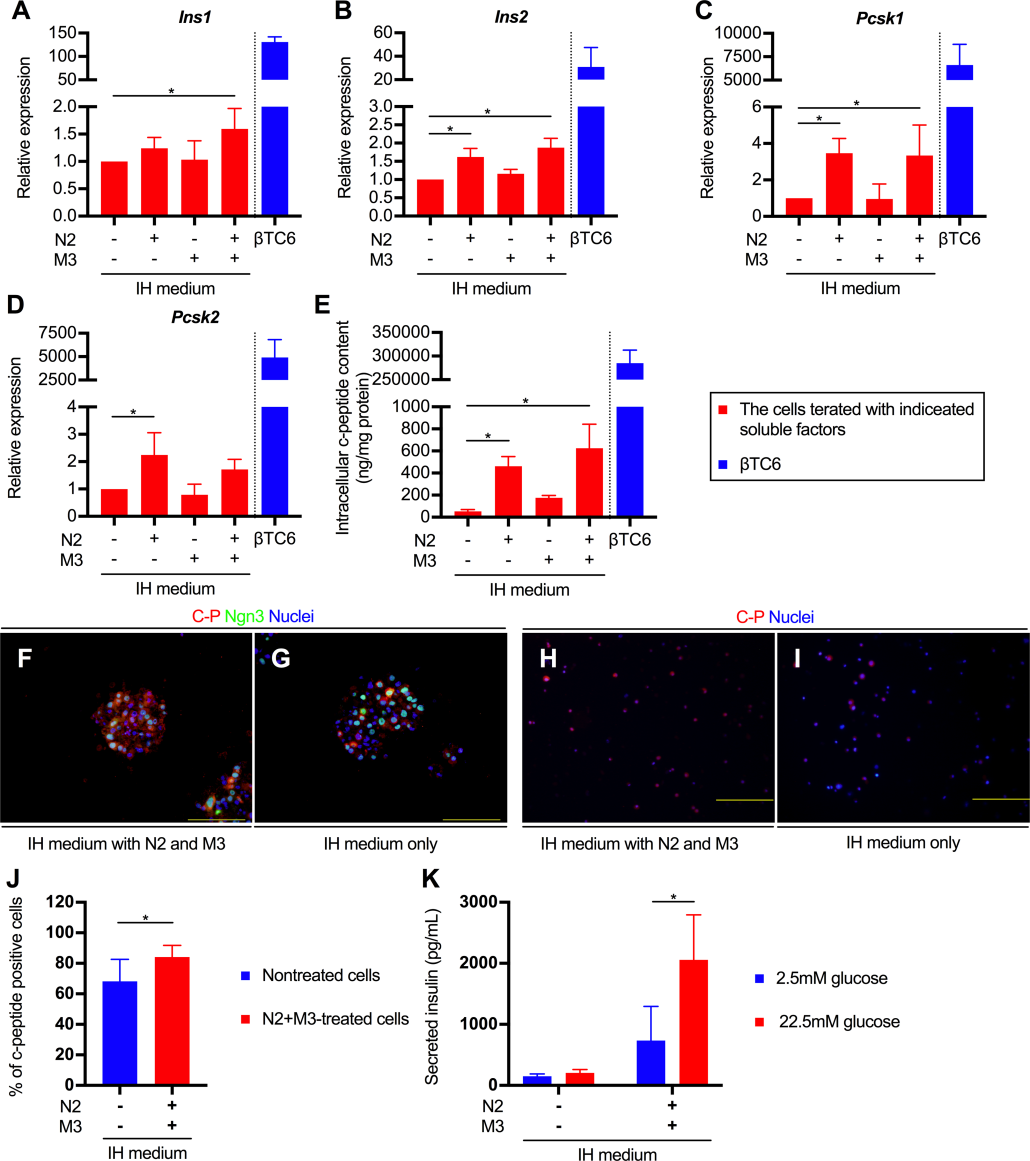
The effect of simultaneous administration of N2 and M3 for cellular transdifferentiation. (A, B, C and D) The expression of *Ins1* (A), *Ins2* (B), *Pcsk1* (C), and *Pcsk2* (D) of infected cells treated with indicated supplements. The data are presented as fold-changes in the gene expression levels relative to the values in the untreated cells (n = 4). M3 contained exendin-4, L685,458, and noggin. IH medium indicates the basal medium composed of IMDM and Hamm's F12. The detailed composition is listed in Table 1. *P < 0.05 (one-way ANOVA followed by Dunnett’s test. The values in the untreated cells were used as control.); error bars, S.D. (E) Intracellular c-peptide contents in infected cells treated with indicated supplements (n = 4). *P < 0.05 (one-way ANOVA followed by Dunnett’s test. The value of untreated cells was used as a control.); error bars, S.D. (F and G) Immunostaining with c-peptide and Ngn3 antibody for transdifferentiated cells treated with N2 and M3 (F) or without (G). Scale bars, 200 μm. (H and I) Immunostaining with c-peptide antibody in dispersed single-cells from cellular aggregates treated (H) or not treated (I) with N2 and M3. Scale bars, 200 μm. (J) Measurement of the percentage of c-peptide-positive cells in the presence or absence of N2+M3 treatment (n = 5 independent experiments). *P < 0.05 (*t*-test); error bars, S.D. (K) Insulin secretion by transdifferentiated cells in response to the indicated concentrations of glucose (n = 4). *P < 0.05 (*t*-test); error bars, S.D. The results obtained from the βTC6 cells were excluded from all the statistical analyses, because of the marked deviations from other values. N2, N2 supplement; *Pcsk1*, proprotein convertase subtilisin/kexin type 1; *Pcsk2*, proprotein convertase subtilisin/kexin type 2; C-P, c-peptide; Ngn3, neurogenin 3.

Based on these results, we further examined the necessity of N2 and M3 individually. As shown in Fig 4A and 4B, the expressions of both *Ins1* and *Ins2* were greatly enhanced when the cells were treated with N2 plus M3. We further analyzed the expression of the proprotein convertase subtilisin/kexin type 1 (*Pcsk1*) and type2 (*Pcsk2*) genes, which encode proprotein convertase 1/3 (PC1/3) and 2 (PC2), respectively. In mature β cells, both PC1/3 and PC2 are involved in the process of generation of insulin and c-peptide from proinsulin. In the predominant processing pathway, proinsulin is first converted to the intermediate des-31,32-proinsulin following cleavage by the PC1/3, and subsequently to insulin and c-peptide following cleavage by the PC2 [18]. As a result, the expressions of both *Pcsk1* and *Pcsk2* were enhanced by N2+M3 treatment; in particular, expression of the former was significantly enhanced as compared with that in the untreated cells (Fig 4C and 4D). These results indicate that the concurrent administration of N2 and M3 might generate insulin-producing cells in which prohormone processing similar to that in mature β cells occurs. Indeed, the intracellular c-peptide contents of cells treated with N2 and M3 was increased by 12-fold, compared with that in untreated cells (Fig 4E). The relative expression levels of *Ins1*, *Ins2*, and c-peptide protein in the N2+M3-treated, transfected PBLHCs were 1/80^th^, 1/17^th^, and 1/450^th^, respectively, as compared with the corresponding values in the βTC6 cells. Furthermore, an immunofluorescent analysis revealed the emergence of c-peptide-positive cellular aggregates and the cytoplasmic localization of c-peptide protein (Fig 4F and 4G), and the emergence of c-peptide positive cells was significantly increased by administration of N2 and M3 compared to the untreated control group (84.1 ± 7.6% vs. 68.3 ± 14.3%, respectively; *P* < 0.001) (Fig 4H, 4I and 4J). To examine the functionality of transdifferentiated insulin-producing cells, we measured the insulin level in the culture medium after incubation with glucose. While there was no significant change in insulin secretion of untreated cells even when high glucose was applied, the treated cells secreted insulin in a glucose-dependent manner (Fig 4K). These results suggest that the N2 and M3 treatment not only contributed to upregulation of the relevant gene expressions in the TF-mediated-transdifferentiated insulin-positive cells, but also to their ability to secrete insulin in response to glucose stimulation.

### Expressions of various pancreatic β cell-specific genes were enhanced by N2+M3 treatment

**Fig 5:**
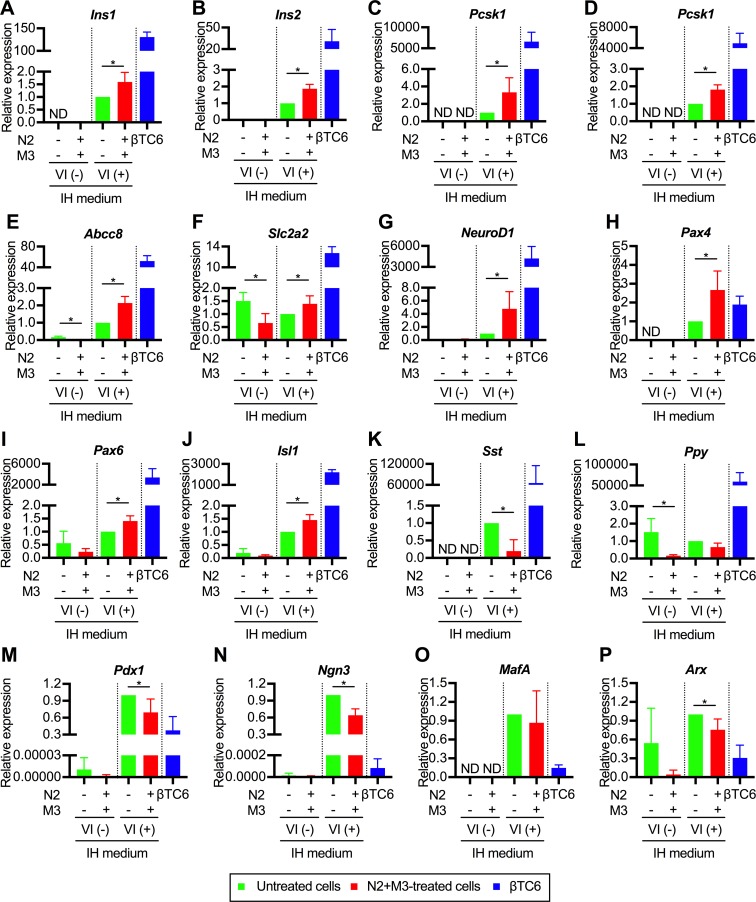
Expressions of pancreas-related genes in the transdifferentiated cells. The data are presented as the fold-change in gene expression relative to the value in the transfected cells not treated with N2+M3 (n = 4). M3 contained exendin-4, L685,458, and noggin. IH medium indicates the basal medium composed of IMDM and Hamm's F12. The detailed composition is listed in Table 1. *P < 0.05 (*t*-test or Mann-Whitney U test, as appropriate); error bars, S.D. The results obtained from the βTC6 cells were excluded from all the statistical analyses, because of the marked deviations from other values. N2, N2 supplement; VI, viral infection; *Ins1*, insulin I; *Ins2*, insulin II; *Pcsk1*, proprotein convertase subtilisin/kexin type 1; *Pcsk2*, proprotein convertase subtilisin/kexin type 2; *Abcc8*, the ATP-binding cassette sub-family C member 8; *Slc2a2*, solute carrier family 2 member 2; *NeuroD1*, neurogenic differentiation 1; *Pax4*, paired box gene 4; *Pax6*, paired box gene 6; *Isl1*, islet 1; *Sst*, somatostatin; *Ppy*, pancreatic polypeptide; *Pdx1*, pancreas duodenal homeobox 1; *Ngn3*, neurogenin 3; *MafA*, v-maf avian musculoaponeurotic fibrosarcoma oncogene homolog A; *Arx*, aristaless related homeobox.

To elucidate the detailed mechanisms involved in the effects of N2+M3 treatment for pancreatic transdifferentiation, we analyzed the expressions of various pancreas- and liver-related genes with stratification in both transfected and non-transfected cells. Expressions of various pancreas-related genes were up-regulated in the transfected cells treated with N2+M3 as compared to the untreated cells. The significantly enhanced gene expressions included not only those of insulin (Fig 5A and 5B) and prohormone convertase genes (Fig 5C and 5D), but also of those encoding proteins involved in insulin secretion (ATP-binding cassette sub-family C member 8 [*Abcc8*, or so-called sulfonylurea receptor 1], solute carrier family 2 member 2 [*Slc2a2*, or so-called glucose transporter 2]) (Fig 5E and 5F), and endogenous pancreatic TF genes (*NeuroD1*, paired box gene 4 [*Pax4*], paired box gene 6 [*Pax6*], and islet 1 [*Isl1*]) (Fig 5G, 5H, 5I and 5J). In contrast, the expressions of genes encoding pancreatic hormones other than insulin (*Sst* and *Ppy*, Fig 5K and 5L) were not up-regulated by N2+M3 treatment. Among the pancreas-specific TF genes, although the expressions of *Pdx1*, *Ngn3* and *MafA* were not up-regulated by aforementioned treatment, the expression levels were by themselves higher than those in the βTC6 cells (Fig 5M, 5N and 5O). The expression of aristaless related homeobox (*Arx*) gene, which was instrumental for the allocation to the pancreatic α-cell lineages [19], was significantly down-regulated in N2 and M3-treated cells (Fig 5P). The expressions of *Gcg* and the NK6 transcription factor related, locus 1 (*Nkx6*.*1*) gene were not observed regardless of the administration of N2 and M3.

**Fig 6:**
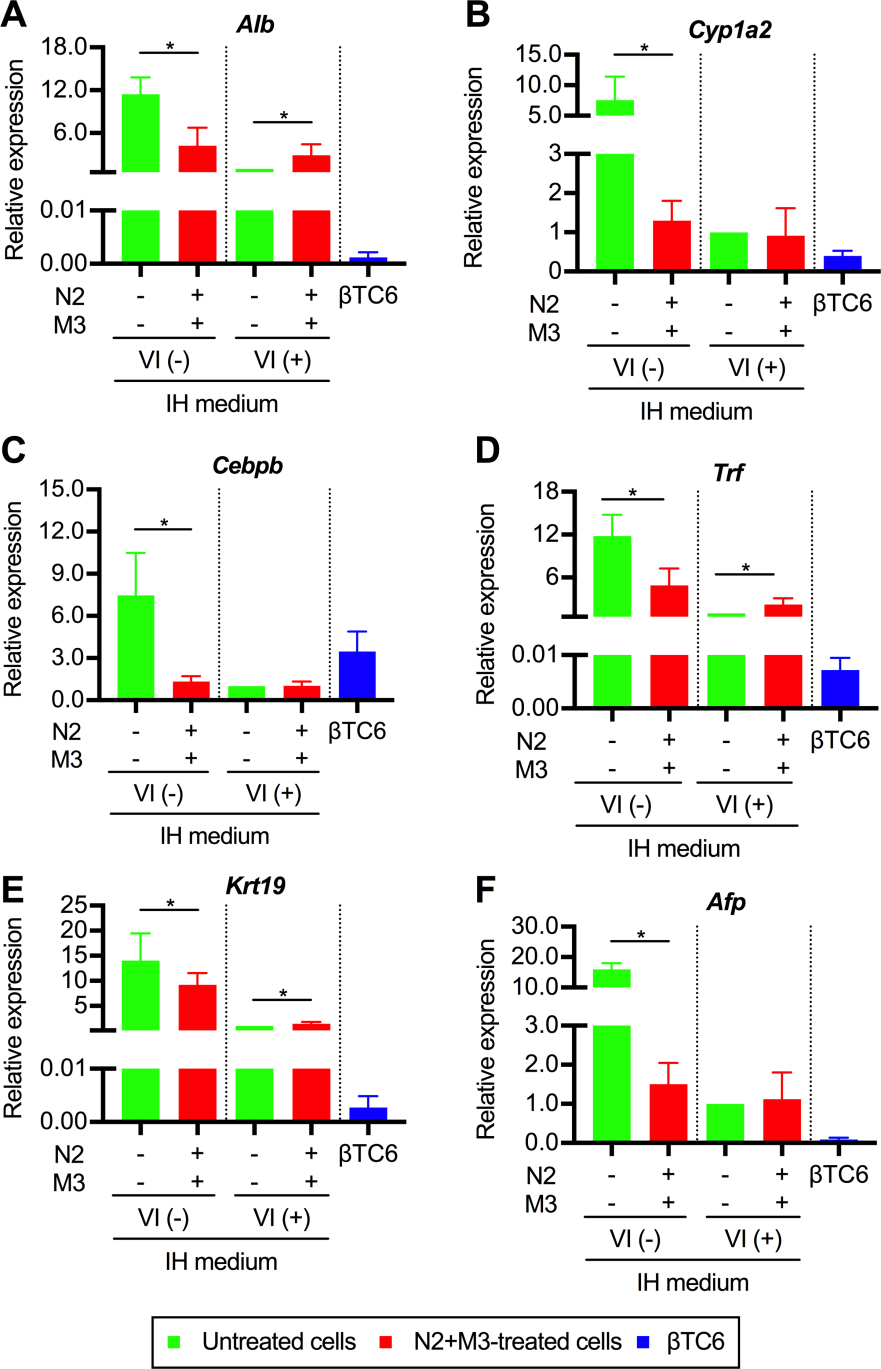
The gene expressions of liver-related genes in the transdifferentiated cells. The data are presented as fold-changes in the gene expression levels relative to the values of the transfected cells not treated with N2+M3 (n = 4). M3 contained exendin-4, L685,458, and noggin. IH medium indicates the basal medium composed of IMDM and Hamm's F12. The detailed composition is listed in Table 1. *P < 0.05 (*t*-test or Mann-Whitney U test, as appropriate); error bars, S.D. The results obtained from the βTC6 cells were excluded from all the statistical analyses, because of the marked deviations from other values. N2, N2 supplement; VI, viral infection; *Alb*, albumin; *Cyp1a2*, cytochrome P450 1A2; *Cebpb*, CCAAT/enhancer-binding protein β; *Trf*, transferrin; *Krt19*, keratin 19; *Afp*, alpha-fetoprotein.

As for the expressions of the liver-related genes, since PBLHCs are bipotent liver progenitor cells, we individually analyzed for hepatocyte- or cholangiocyte-related, and fetal liver-specific genes. The following six genes were actually analyzed: albumin (*Alb*) (Fig 6A); cytochrome P450 1A2 (*Cyp1a2*) (Fig 6B); CCAAT/enhancer-binding protein β (*Cebpb*) (Fig 6C); transferrin (*Trf*) (Fig 6D); keratin 19 (*Krt19*) (Fig 6E); and alpha-fetoprotein (*Afp*) (Fig 6F). The results revealed that the expressions of all of the six aforementioned genes were significantly downregulated in the non-transfected cells treated with N2+M3. On the other hand, the downregulation of the gene expressions associated with N2+M3 treatment could not be confirmed in the transfected cells. These results indicate that N2+M3 treatment may be involved in the hepatic dedifferentiation process.

### N2 and M3-treated transdifferentiated cells ameliorate hyperglycemia in diabetic mice

**Fig 7:**
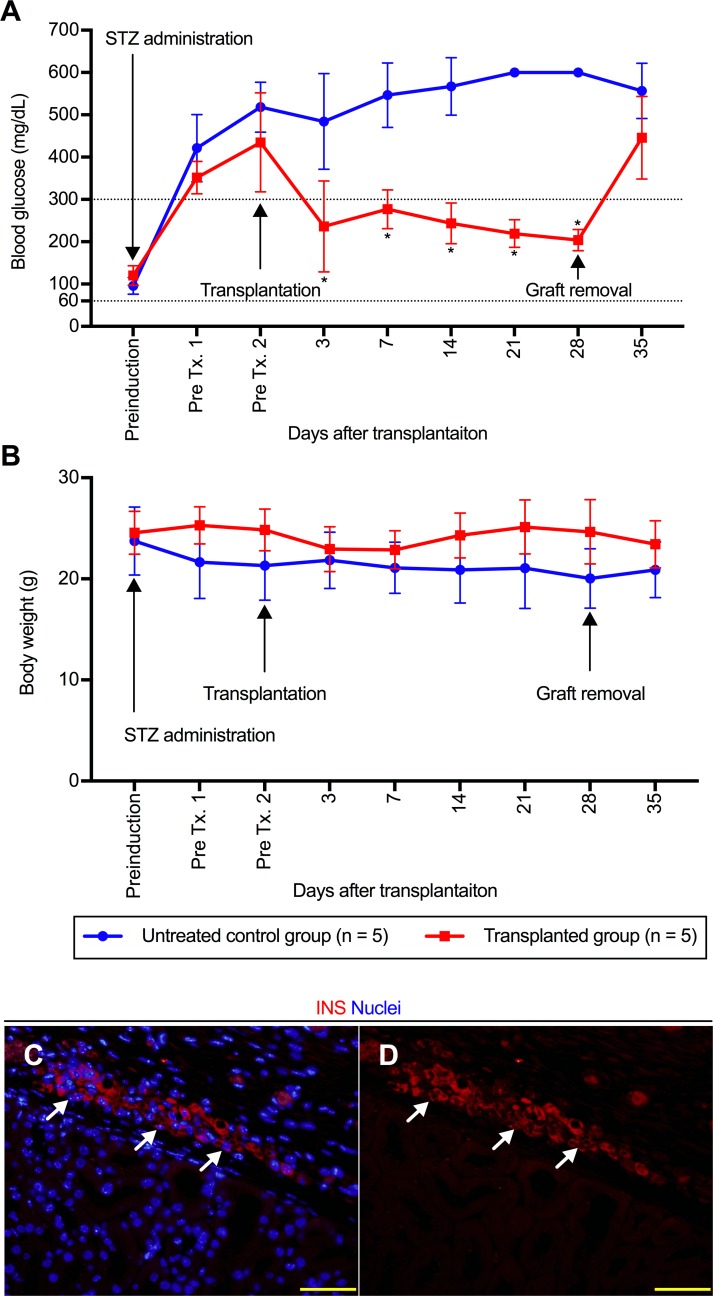
Transdifferentiated cells ameliorate hyperglycemia in diabetic mouse. (A and B) The non-fasting blood glucose levels (A) and body weight (B) in diabetic mice. The red line with squares indicates the values obtained from the mice transplanted with N2 and M3-treated transdifferentiated cells (n = 5), and the blue line with circles indicates those obtained from the untreated control mice (n = 5). M3 contained exendin-4, L685,458, and noggin. *P < 0.05 (*t*-test); error bars, S.D. (C and D) Immunostaining with insulin antibody for transplanted transdifferentiated cells treated with N2 and M3. Arrow, insulin-positive cells. Scale bar, 50 μm. STZ, streptozotocin; Tx, transplantation; N2, N2 supplement; INS, insulin.

The functionality of N2 and M3-treated transdifferentiated cells was further analyzed *in vivo* by transplanting the treated cellular aggregates into the kidney subcapsule in immunodeficient mice. The average number of transplanted aggregates was 1036 ± 405/body. Although the number of transplanted cells was not calculated at the time of transplantation, it was estimated to be approximately 3.8 ± 0.6 × 10^6^/body, based on measurement of the aggregates prepared under the same conditions. As noted in Fig 7A, the transplantation of treated cells into diabetic mice resulted in a statistically significant amelioration of hyperglycemia at 3 days after transplantation. The reversal of hyperglycemia was maintained until a nephrectomy at 28 days after transplantation. After graft removal, the blood glucose values of each animal were immediately elevated and reached levels similar to those observed in untreated control animals. In regard to the changes in the body weights of the diabetic mice, the average body weight in the untreated control group at each time-point was low, although there were no statistically significant intergroup differences (Fig 7B). The presence of insulin-positive cells was observed in renal subcapsular space of removed graft (Fig 7C and 7D). Although one mouse in the untreated control group died at 15 days after the diagnosis of diabetes, all the other mice survived for the entire study period. To compare the functionality of the transdifferentiated cells with that of cells actually having insulin-secreting ability, we created the βTC6-transplanted model (n = 5) (S2A and S2B Fig). Consistent with the findings in the N2+M3-treated cell-transplanted model, improvement of hyperglycemia was noted in all individuals on day 3 after transplantation of the βTC6 cells. However, the blood glucose levels in these mice continued to decline, and on day 14 after transplantation, 3 out of the 5 mice developed hypoglycemia and had to be sacrificed. One mouse in the βTC6-transplanted group died on day 21 after the transplantation. Left nephrectomy performed on day 28 after the transplantation in 1 out of the 5 mice which showed an amelioration of hyperglycemia immediately resulted in hyperglycemia. These *in vivo* data indicated that the transplantation of N2+M3-treated cells ameliorated the elevated blood glucose levels in diabetic animals. Thus, the results of both *in vitro* and *in vivo* functional analyses revealed that the simultaneous administration of N2 and M3 contributed to the promotion of TFs-mediated liver-to-pancreas functional transdifferentiation.

### TF-mediated pancreatic transdifferentiation could be sustained *in vitro* for a long period by continuous N2+M3 treatment

**Fig 8:**
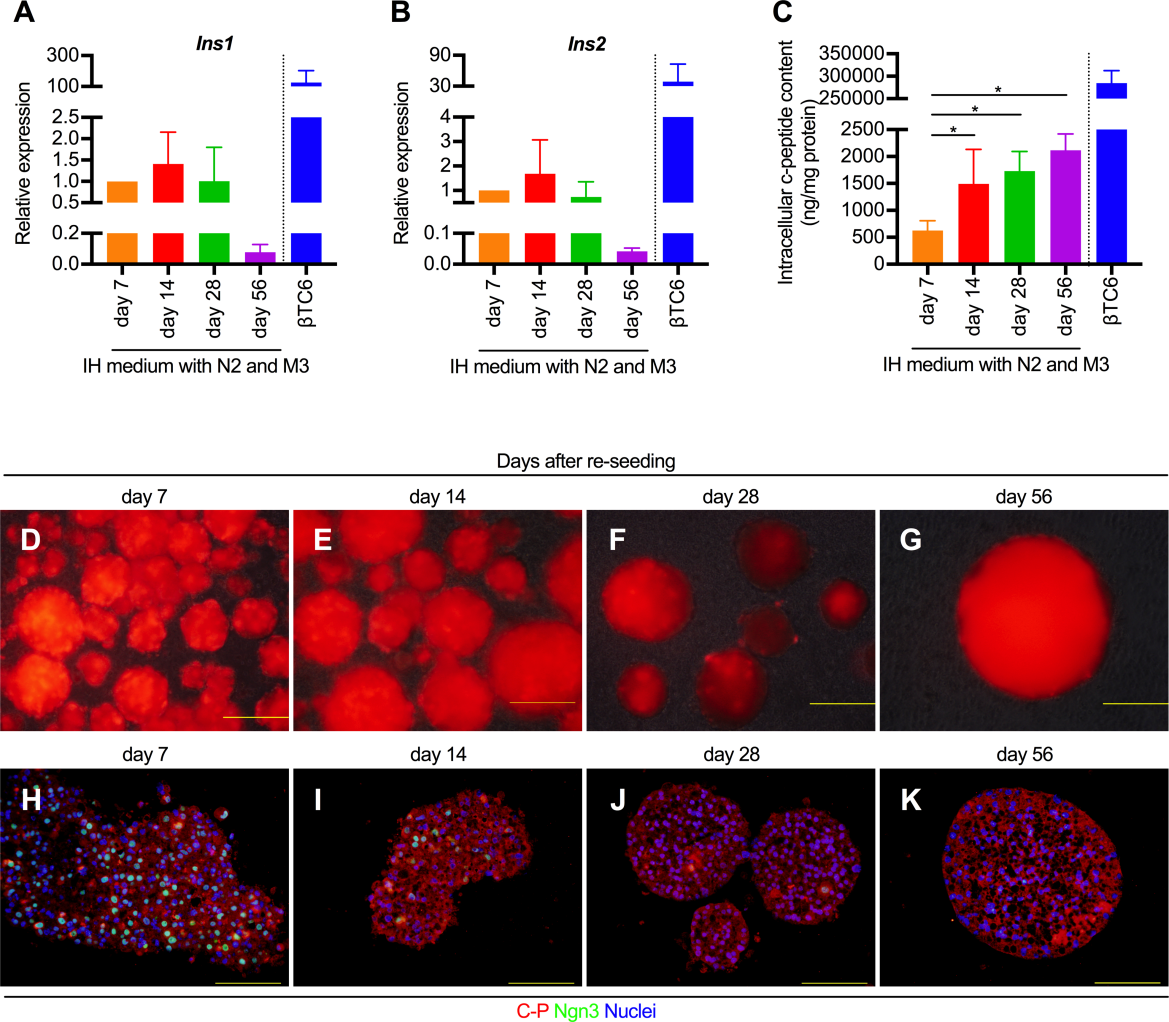
Analysis of the time-course of changes in the insulin-producing ability of the N2+M3-treated transdifferentiated cells. (A and B) The expression levels of *Ins1* (A) and *Ins2* (B) in the N2+M3-treated transdifferentiated cells at different time-points. The data are presented as fold-changes in the gene expression levels relative to the values in the cells measured on day 7 days after re-seeding (n = 4). M3 contained exendin-4, L685,458, and noggin. IH medium indicates the basal medium composed of IMDM and Hamm's F12. The precise composition is listed in Table 1. *P < 0.05 (one-way ANOVA followed by Dunnett’s test. The values in the cells on day 7 days after re-seeding were used as the control.); error bars, S.D. (C) Intracellular c-peptide contents in the N2+M3-treated transdifferentiated cells at different time-points (n = 4). *P < 0.05 (one-way ANOVA followed by Dunnett’s test. The values in the cells on day 7 after re-seeding were used as the control.); error bars, S.D. (D to K) Representative microscopic images (D, E, F, and G) and immunostaining of the transdifferentiated cells treated continually with N2 and M3 for c-peptide and Ngn3 (H, I, J, and K) at four different time-points. The results obtained from βTC6 were excluded from all the statistical analyses, because of the marked deviations from other values. N2, N2 supplement; C-P, c-peptide; Ngn3, neurogenin 3.

Finally, in order to ascertain how long the transdifferentiated cells maintained their insulin-producing ability, we cultured these cells in the presence of N2+M3 until 56 days after re-seeding, and compared the insulin gene expressions and intracellular c-peptide contents among different time-points. The results revealed that the gene expression levels of both *Ins1* and *Ins2* peaked at 14 days after re-seeding, decreasing gradually thereafter. The expression level of *Ins1* and *Ins2* on day 56 after re-seeding was about 1/15^th^ and 1/25^th^ of those at 7 days, respectively (Fig 8A and 8B). On the other hand, the intracellular c-peptide content was maintained at the similar level from day 14 to day 56 days after re-seeding (Fig 8C). Fig 8D to 8K show the time-dependent changes in the transdifferentiated cells with continual N2+M3 treatment. Although the expressions of mCherry fluorescence and c-peptide were maintained until day 56 after re-seeding, the expression of exogenous Ngn3 began to decrease gradually from day 7 after re-seeding, and its expression could not be confirmed on day 56. These results indicate that the insulin-producing ability of the transdifferentiated cells can be sustained *in vitro* for a long period (at least 1 month) by continuous N2+M3 treatment.

## Discussion

Liver-derived cells can be transdifferentiated into insulin-producing cells through a process mediated by the ectopic expression of TFs, and the resulting cells could potentially be used to treat diabetes [8, 20, 21]. Although the types of TFs that should be introduced have been intensively explored, little attention has been paid to optimizing the culture conditions for infected cells to obtain phenotypic and functional maturation. We herein demonstrated that the TFs-mediated pancreatic transdifferentiation of liver-specific progenitors (PBLHCs) was influenced by extracellular factors, namely, a microenvironment containing specific soluble factors. The significance of our study was emphasized by the following two aspects: i) the usability of PBLHCs as a cell source for transdifferentiation, and ii) the acceleration of functional maturation of transdifferentiated cells in response to the inclusion of known soluble factors.

Several transdifferentiated insulin-producing cells from various cell sources have been reported [22]. Liver cells, in particular, are thought to be promising candidates because the proliferative capacity of the liver depends on tissue-specific progenitors [23]. In general, however, these progenitors are thought to emerge in damaged liver after the administration of a hepatotoxin [24, 25], and specific genetic modifications are required to isolate these cells from normal liver [26]. In contrast, PBLHCs can be efficiently induced by modifying the portal blood flow through portal branch ligation or embolization [11]. Since portal branch ligation or embolization is already performed as a clinical application to increase the volume of the future liver remnant in patients undergoing an extended hepatectomy, the safety of this procedure has been confirmed in clinical settings [27–29]. Another advantage of hepatic cells as a cell source for transdifferentiation is the fact that the liver and the pancreas are developmentally related. In embryonic development, the pancreas and liver share a developmental history up to the stage of bud formation [30]. Furthermore, the ability of cells from different origins to give rise to insulin-producing cells has been shown to depend on their existing developmental relatedness to pancreatic β cells [31]. Recently, Banga and colleagues revealed that Sox9-positive cells existing in association with small bile ducts in the periportal region were potential cell-source candidates for *in vivo* TFs-mediated transdifferentiation in the liver [32]. Since PBLHCs are also positive for Sox9, the *in vivo* administration of a TFs-expressing adenoviral vector in a portal branch-ligated liver lobe *in situ* could be a feasible and innovative procedure for safe and efficient liver-to-pancreas transdifferentiation without requiring either cellular transplantation or immunosuppression.

In estimating the significance of the N2+M3 treatment, it is important to determine whether this treatment mainly improves the transdifferentiation efficiency of the cells or promotes functional maturation of the cells after transdifferentiation. As shown in Figs 5C, 5D and 4E, we found significant increase of the intracellular c-peptide contents and *Pcsk* gene expressions in the cells treated with N2 and M3, as compared to the cells not treated with N2 and M3. Furthermore, we also showed that only the cells treated with N2 and M3 acquired glucose-dependent, insulin-secreting ability. The expressions of various pancreas-related genes were also upregulated by simultaneous treatment of the cells with the administration of the aforementioned soluble factors. These findings appear to suggest that N2+M3 treatment has the effect of inducing further maturation of transdifferentiated cells into cells that can produce and secrete insulin. On the other hand, there is little direct evidence to prove that N2+M3 treatment promotes liver-to-pancreas transdifferentiation per se. As shown in Fig 4J, the percentage of c-peptide positive cells increased in the cells treated with soluble factors as compared to the non-treated cells, but this can also be regarded to be a result of the aforementioned promotion of functional maturation. In stratified analyses based on the presence or absence of gene transfer, the expressions of liver-related genes were comprehensively attenuated by N2+M3 treatment in the non-transfected cells, suggesting that N2+M3 treatment may be involved in the hepatic dedifferentiation process, although similar findings could not be confirmed in the transfected cells. Therefore, we speculate that N2+M3 treatment mainly contributes to functional maturation of the cells after transdifferentiation. In regard to the effect of N2+M3 treatment *per se* in promoting transdifferentiation, further investigation of the influence of soluble factors on hepatic dedifferentiation, including the expressions of liver-related genes, is necessary.

We focused on the functional maturation of TFs-mediated transdifferentiated cells by known soluble factors in combination with a serum-free medium because of the following advantages: i) the ability to provide adequate signals that accelerate pancreatic transdifferentiation; ii) the exclusion of potential inhibitors of differentiation, including serum; and iii) the avoidance of the risk of contamination with pathogens that could lead to human health risks. Zalzman et al. investigated in detail the effect of activin A, a cytokine belonging to the TGF-β superfamily, on transdifferentiation [21]. They used immortalized fetal human liver progenitor cells transduced with lentiviral Pdx1, designated as FH-B-TPN cells, and analyzed the efficiency of transdifferentiation depending on the presence or absence of activin A. As a result, the intracellular insulin content of FH-B-TPN cells treated with activin A was found to be almost 4-fold higher than that in untreated cells. Furthermore, the insulin content of cells supplemented with activin A in combination with serum-free medium was greatly increased to 33 times that of cells grown in serum-containing medium. Although Zalzman et al.’s study could not reveal the detailed mechanisms of activin A on the acceleration of transdifferentiation, the present results suggest that TFs-mediated transdifferentiation is facilitated by the presence of adequate molecules and that these molecules synergistically act in combination with serum-free medium to induce transdifferentiation.

In this study, we showed that the combined administration of N2 and M3 (exendin-4, L-685,458 and noggin) was the accelerator of functional maturation of TFs-mediated transdifferentiated cells. We wish to emphasize that these factors have been used in the process of endocrine lineage commitment in reported protocols for generating pancreatic endocrine cells from stem cells [33]. Although the pathways regulating the commitment and maturation of endocrine lineages from pancreatic progenitors are not completely understood, endocrine lineage commitment is thought to be achieved in the presence of bone morphologic protein (BMP), TGF-β/activin/nodal, and notch inhibition [34]. Noggin is an inhibitor of TGF-β superfamily signaling, inhibiting the BMP pathway by acting as an antagonist [35]. Since BMP inhibition is required for dorsal-ventral endoderm patterning in normal embryogenesis [36, 37], previous protocols have included noggin during the induction of Pdx1-positive pancreatic progenitors [38–40]. Recently, Nostro and colleagues revealed that BMP signaling plays a key role in determining whether the foregut endoderm cells adopt a ventral pancreatic or hepatic fate; thus, the inhibition of BMP signaling was shown to be essential for pancreatic development [33]. Although TGF-β superfamily signaling plays a critical role in the regulation of cell growth, differentiation, and the development of multiple organ systems including the pancreas [41], the absence of this pathway caused by the pancreas-specific deletion of SMAD4, which is a common intracellular signaling transducer of the TGF-β superfamily, had no discernable impact on pancreatic development [42]. Based on this observation, Nostro et al. also revealed that the simultaneous inhibition of the TGF-β/activin/nodal and BMP pathways during endocrine lineage commitment led to increases in the levels of insulin expression and cellular proliferation. Furthermore, independent analyses of each pathway showed that the inhibition of BMP signaling was primarily responsible for the increase in insulin expression, whereas the inhibition of TGF-β/activin/nodal signaling led to an increase in cell numbers, likely promoting the survival and expansion of the newly formed endocrine cells [33]. The notch inhibitor L-685,458 is also included in hiPSC differentiation cultures following the induction of pancreatic epithelium [33], based on the following observations: Ngn3 *per se* induces the differentiation of undifferentiated pancreatic epithelial cells toward mature islet cells, and the transcriptional activity of the Ngn3 promoter is inhibited via the notch pathway [43]. Thus, the activation of notch signaling arrests pancreatic differentiation at the endocrine progenitor stage [44–46]. Exendin-4 is a glucagon-like peptide-1 (GLP-1) receptor agonist. GLP-1 is produced in enteroendocrine L cells of the gut and acts as a glucose regulatory hormone mediated by pancreatic gene expression including insulin, prohormone production, and glucose-regulated insulin secretion. Moreover, GLP-1 induces β cell proliferation, neogenesis, and survival [47, 48]. Based on these bioactivities, GLP-1 and exendin-4 are utilized in several pancreatic differentiation protocols from stem cells [1, 38, 49, 50]. Furthermore, Aviv and colleagues demonstrated that exendin-4 exerts biological effects that are similar to those of GLP-1 even in Pdx1-mediated, transdifferentiated hepatic cells [51]. Although further investigations are needed to verify whether the action mechanisms via the aforementioned soluble factors, especially developing pancreas-specific signaling cascades, are involved in liver to pancreas transdifferentiation, the presently reported results support the idea that specific soluble factors that are capable of promoting functional maturation of TFs-mediated transdifferentiated cells may exist.

Both N2 and B27 supplements have been widely used in protocols for generating pancreatic endocrine cells from hiPSCs/human embryonic stem cells (hESCs) [1, 16, 33]. Although these supplements were developed as serum replacement components in serum-free primary cultures of neural cells, the composition of each is largely different. Specifically, five molecules are included in the former [52], while the latter contains 20 kinds of molecules [53]. Therefore, it is speculated that the latter may contain some factors that inhibit the induction of transdifferentiation. Likewise, regarding the components of the former, i.e., insulin, transferrin, selenite, progesterone, and putrescine, it will be necessary to examine the effects of each of these components on transdifferentiation individually.

As shown in Fig 4, although the expression levels of the insulin genes increased by approximately 1.5- to 2-fold following N2+M3 treatment as compared to the levels in the untreated cells, the intracellular c-peptide content in the N2+M3-treated cells increased by approximately 12-fold as compared to that in the untreated cells. These results indicate a divergence between the expressions of the insulin genes and c-peptide protein. One possible explanation for the aforementioned discrepancy is the possible difference in the proinsulin processing activity of proprotein convertase (PC) depending on the presence or absence of N2+M3. Proinsulin is synthesized in the pancreatic β cells and subsequently cleaved by two processing endopeptidases, PC1/3 and PC2, to release insulin and c-peptide in the secretory granules of the pancreatic β cells [18]. In our experiments, the expressions of both *Pcsk1* and *Pcsk2*, which encode PC1/3 and PC2, respectively, were enhanced in the N2+M3-treated cells as compared to the untreated cells. Since the c-peptide detection kit used by us (Mouse C-peptide ELISA Kit, Shibayagi) was specific for the mouse c-peptide, neither proinsulin nor insulin could be detected with the kit. Therefore, it is assumed that the difference in the intracellular c-peptide content depending on the presence or absence of N2+M3 may be higher than that in the insulin gene expression level. Indeed, even in the cells treated with only N2, which showed significantly enhanced expressions of both *Pcsk1* and *Pcsk2*, the extent of increase in the intracellular c-peptide content was confirmed to be nearly the same as that in the N2+M3-treated cells. Likewise, since the c-peptide antibody we used was reactive with proinsulin, we speculate that the difference in the c-peptide-positive cell ratio was small between the N2+M3-treated and non-treated cells because the proinsulin in the cells was also stained. In addition to the above, analyses of the time-course of changes in the expression levels of the insulin genes and c-peptide content in the N2+M3-treated cells revealed that while the insulin gene expressions in the cells decreased by day 56 days after re-seeding, the intracellular c-peptide content was maintained. Although we are unable at present to provide a clear explanation for this finding, it is possible that the c-peptide protein accumulated in the cytoplasm during the prolonged culture period. It could also be related to the fact that the half-life of c-peptide in the serum is longer than that of insulin [54].

In summary, we have demonstrated that infected PBLHCs can be induced to transdifferentiate into insulin-producing and secreting cells using a 3D culture system in the presence of appropriate soluble factors. Although the mechanisms underlying the effects of the molecules used in this study to promote cellular functional maturation remain uncertain, the present results suggest that if the *in vivo* manipulation of genetic factors is combined with molecules targeting specific pathways, the human liver could become an ideal source organ for obtaining functional insulin-producing cells. Furthermore, the aforementioned TFs-mediated transdifferentiation of cells in the liver *in situ* could likely be accelerated with the use of portal vein ligation or embolization. Although additional studies are needed to refine our protocol, this study provides an important strategy for the cellular transdifferentiation of adult somatic cells using defined factors and suggests a potentially useful therapeutic option for curing patients with diabetes.

## Supporting information

S1 Fig Exogenous co-expression of transcription factors (TFs) achieved by co-infection with the appropriate adenovirus vectors and endogenous expressions of the genes encoding pancreatic hormones in portal branch ligation-stimulated hepatic cells (PBLHCs). (A and B) Immunostaining for co-infected PBLHCs revealed the nuclear localization of each ectopically expressed TF and nuclear localized GFP. PNM and PDM represent co-infection of three adenoviral vectors (pAd-Pdx1 and pAd-MafA with either pAd-Ngn3 or pAd-NeuroD1), respectively. Scale bars, 200 μm. (C and D) Semiquantitative RT-PCR analyses stratified according to the combination of TFs that were used were performed using primer sets for insulin (C) and other pancreatic hormones (D). “Pre” represents PBLHCs without viral infection. *Hprt* was used as an internal control.
(TIF)

S2 Fig Changes in the blood glucose levels and body weights of βTC6-transplanted diabetic mice. (A and B) The non-fasting blood glucose levels (A) and body weights (B) of the βTC6-transplanted diabetic mice. Three out of the 5 mice developed hypoglycemia and had to be sacrificed on day 14 after the transplantation. One mouse of this group died on day 21 after the transplantation. Left nephrectomy performed on day 28 after the transplantation in 1 out of the 5 mice which showed amelioration of hyperglycemia immediately resulted in hyperglycemia. STZ, streptozotocin; Tx, transplantation.
(TIFF)

S1 Table Details of antibodies used for immunohistochemically analysis. (DOCX)

S1 Dataset Data underlying this study. (ZIP)
