# Supplementary figures and images for: Treatment with specific soluble factors promotes the functional maturation of transcription factor-mediated, pancreatic transdifferentiated cells
Source: PLoS One. 2018 May 16;13(5):e0197175. doi: 10.1371/journal.pone.0197175 (PMC5955553; doi:10.1371/journal.pone.0197175)

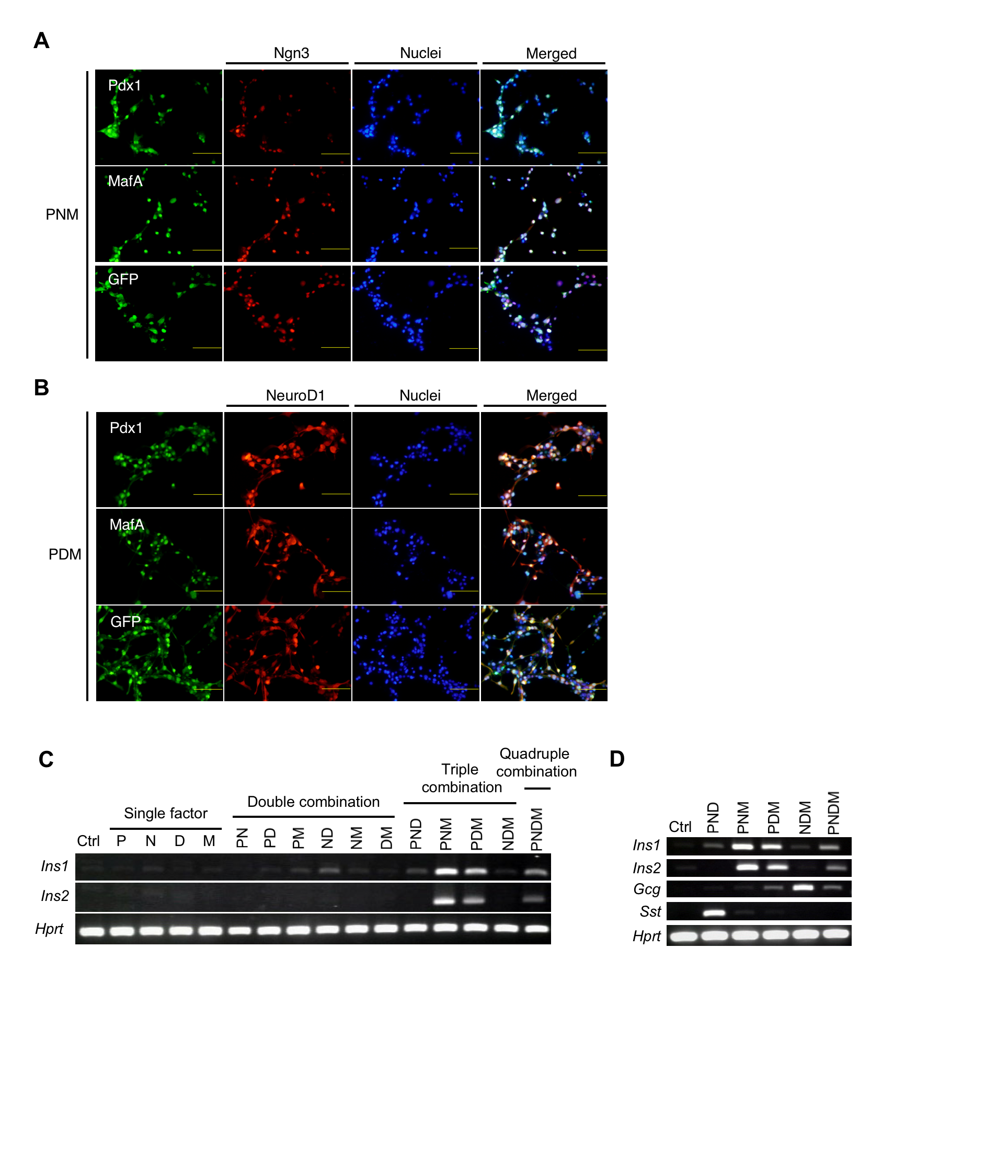

Supplement: S1 Fig — (A and B) Immunostaining for co-infected PBLHCs revealed the nuclear localization of each ectopically expressed TF and nuclear localized GFP. PNM and PDM represent co-infection of three adenoviral vectors (pAd-Pdx1 and pAd-MafA with either pAd-Ngn3 or pAd-NeuroD1), respectively. Scale bars, 200 μm. (C and D) Semiquantitative RT-PCR analyses stratified according to the combination of TFs that were used were performed using primer sets for insulin (C) and other pancreatic hormones (D). “Pre” represents PBLHCs without viral infection. Hprt was used as an internal control. (TIF) [file pone.0197175.s001.tif]

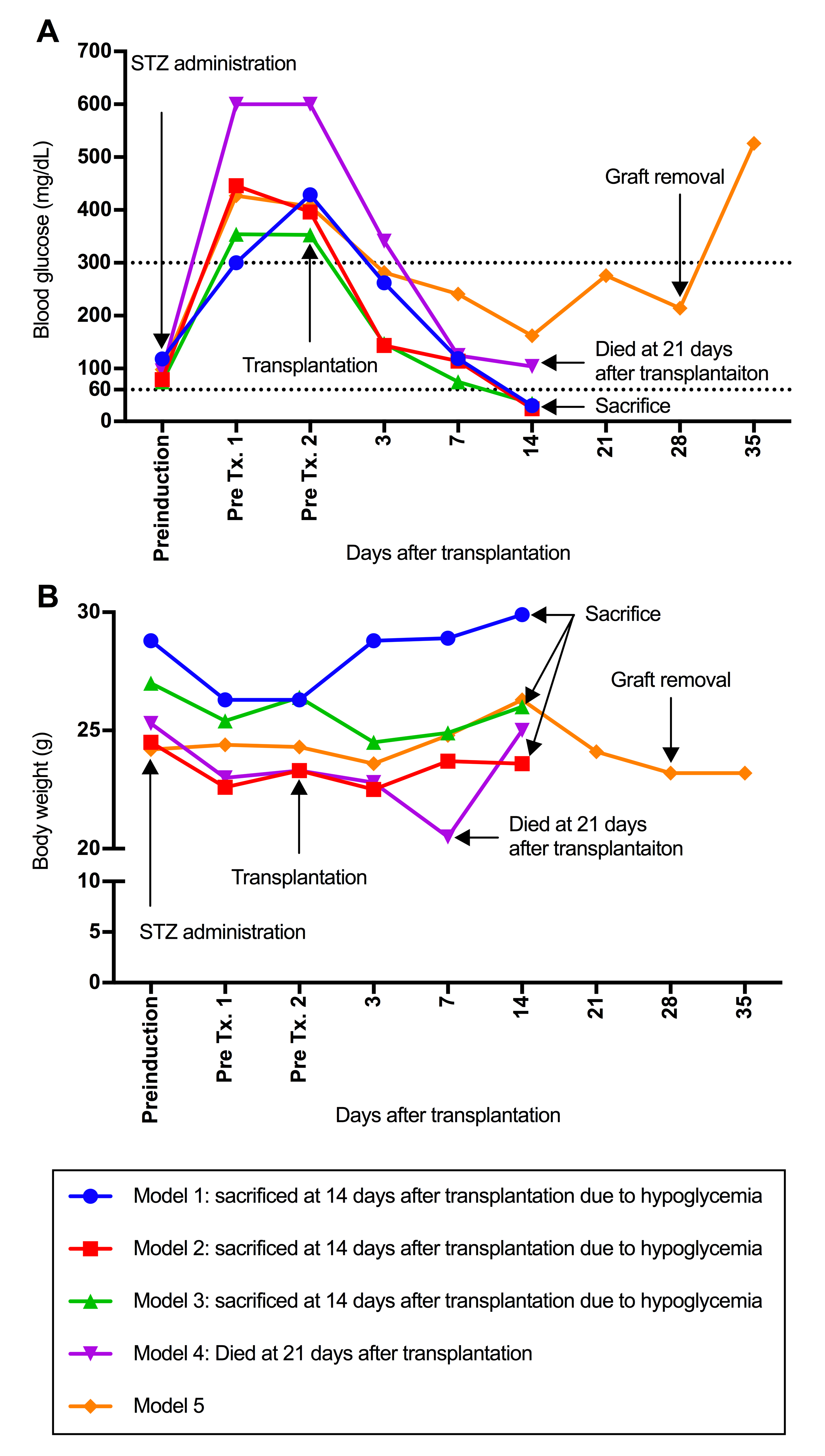

Supplement: S2 Fig — (A and B) The non-fasting blood glucose levels (A) and body weights (B) of the βTC6-transplanted diabetic mice. Three out of the 5 mice developed hypoglycemia and had to be sacrificed on day 14 after the transplantation. One mouse of this group died on day 21 after the transplantation. Left nephrectomy performed on day 28 after the transplantation in 1 out of the 5 mice which showed amelioration of hyperglycemia immediately resulted in hyperglycemia. STZ, streptozotocin; Tx, transplantation. (TIFF) [file pone.0197175.s002.tiff]
