# Supplementary material for: Treatment with specific soluble factors promotes the functional maturation of transcription factor-mediated, pancreatic transdifferentiated cells
Source: PLoS One. 2018 May 16;13(5):e0197175. doi: 10.1371/journal.pone.0197175 (PMC5955553; doi:10.1371/journal.pone.0197175)
Supplement: S1 Table — (DOCX) [file pone.0197175.s003.docx]

| S1 Table. Details of antibodies used for immunohistochemically analysis. | | | | | | | |
| --- | --- | --- | --- | --- | --- | --- | --- |
| Primary antibody | | | |  | Secondary antibody | | |
| Antigen | Host | Dilution | Manufacturer |  | Antibody | Dilution | Company |
| Pdx1 | rabbit | 1:500 | EMD Millipore |  | anti-rabbit IgG Alexa Fluor 488 conjugated | 1:300 | Thermo Fisher Scientific |
| Ngn3 | mouse | 1:1,000 | NICHD Developmental Studies Hybridoma Bank |  | anti-goat IgG Alexa Fluor 555 conjugated | 1:300 | Thermo Fisher Scientific |
| NeuroD1 | goat | 1:250 | R&D Systems |  | anti-mouse IgG Alexa Fluor 488 conjugated | 1:300 | Thermo Fisher Scientific |
| MafA | rabbit | 1:50 | Bethyl |  | anti-mouse IgG Alexa Fluor 555 conjugated | 1:300 | Thermo Fisher Scientific |
| GFP | rabbit | 1:500 | MBL |  | anti-guinea pig IgG Alexa Fluor 555 conjugated | 1:300 | Thermo Fisher Scientific |
| Insulin | guinea pig | 1:500 | Dako |  |  |  |  |
| C-peptide | guinea pig | 1:200 | TaKaRa Bio |  |  |  |  |
| GFP, green fluorescent protein. | | | | | | | |
